# A Liniment for Earache

**Published:** 1886-06

**Authors:** 


**A Liniment for Earache.**—Pavesi recommends a liniment composed of camphorated chloral 2½ parts, pure glycerine 16½ parts, and oil of sweet almonds 10 parts. This is to be well mixed and preserved in a hermetically closed bottle. A pledget of very soft cotton is to be soaked in the liniment and then introduced as far as possible into the affected ear, two applications being made daily. Frictions may also be made each day with the preparation behind the ear. It is claimed that the pain is almost immediately relieved, and even in many cases the inflammation is subdued.

